# A stance dataset with aspect-based sentiment information from Indonesian COVID-19 vaccination-related tweets

**DOI:** 10.1016/j.dib.2023.108951

**Published:** 2023-02-04

**Authors:** Diana Purwitasari, Cornelius Bagus Purnama Putra, Agus Budi Raharjo

**Affiliations:** Department of Informatics Engineering, Institut Teknologi Sepuluh Nopember, Indonesia

**Keywords:** Stance detection, Aspect-based sentiment analysis, Twitter, COVID-19 vaccination

## Abstract

As a platform of social media with high activity, Twitter has seen the discussion of many hot topics related to the COVID-19 pandemic. One such is the COVID-19 vaccination program, which has skeptics in several religious, ethnic, and socioeconomic groups, and Indonesia has one of the largest populations of various ethnicities and religions of countries worldwide. Diverse opinions based on skepticism about the effectiveness of vaccines can increase the number of people who refuse or delay vaccine acceptance. Therefore, it is important to analyze and monitor stances and public opinions on social media, especially on vaccine topics, as part of the long-term solution to the COVID-19 pandemic. This study presents the Indonesian COVID-19 vaccine-related tweets data set that contains stance and aspect-based sentiment information. The data were collected monthly from January to October 2021 using specific keywords. There are nine thousand tweets manually annotated by three independent analysts. We annotated each tweet with three labels of stance and seven predetermined aspects related to Indonesian COVID-19 vaccine-related tweets: *services, implementation*, a*pps, costs, participants, vaccine products*, and *general*. The dataset is useful for many research purposes, including stance detection, aspect-based sentiment analysis, topic detection, and public opinion analysis on Twitter, especially on the policies regarding the prevention of pandemics.

**Specifications Table**

**Table:** 

Subject	Computer Science, Social Science, Natural Language Processing
Specific subject area	Stance Detection on Tweets, Aspect-based Sentiment Analysis, Public Opinion Analysis
Type of data	Text (CSV-formatted)
Method of data acquisition	Twitter API and Annotated by Three Experts.
Data format	Raw, Filtered
Description of data collection	We collected the dataset for specific keywords posted during the ten months from January 2021 to October 2021 and filtered the data for non-Bahasa (Indonesian language), non-COVID-19 vaccination, spam, and duplication. Independent analysts manually labeled the dataset with stance and aspect-based sentiment information. Three analysts, consisting of two researchers in natural language processing (one MSc-level and one BSc-level) and one communication science expert (BSc-level), labeled the sample data. There are three classes of stance and seven predetermined aspects, and each aspect can have a sentiment between positive and negative. The majority voting strategy was used for the final class label.
Data source location	Country: Indonesia
Data accessibility	Repository name: Mendeley Data Data identification number: 10.17632/7ky2jbjwtn.3 Direct URL to data: https://data.mendeley.com/datasets/7ky2jbjwtn/3
Related research article	C.B.P. Putra, D. Purwitasari, A.B. Raharjo, Stance Detection on Tweets with Multi-task Aspect-based Sentiment: A Case Study of COVID-19 Vaccination, *Int. J. Intell. Eng. Syst*. 15 (2022) 515-526. https://doi.org/10.22266/ijies2022.1031.45

**Value of the Data**


 

• These data are valuable for monitoring and analyzing public opinion on Twitter related to the COVID-19 vaccination program during Indonesia's first ten months of the vaccination program, which is helpful as a guide in the development of policies.
• From the *general* COVID-19 vaccination-related tweets, we provided samples of 9,000 tweets that experts labeled with stance and aspect-based sentiment information. Our dataset provides insights into diverse aspects of the COVID-19 vaccination program, making it possible to provide more accurate sentiment information.
• These data concern the opinion of Indonesians toward COVID-19 vaccination and serve as literature for public authorities to detect Indonesian sentiment toward the COVID-19 vaccination policies, which have some skeptics in several social groups. Moreover, these data can be used for many research purposes, including stance detection and aspect-based sentiment analysis, especially public opinion analysis on Twitter.
• This data can help the research community develop state-of-the-art models for stance detection on tweets, especially Indonesian opinion. Moreover, there may be another pandemic with vaccination policies, so the data can be compared and referenced for developing models.

## 1 Objective

The dataset was collected and processed to further increase our understanding of how sentiment and contextual information impact the performance of stance detection on Twitter text (tweet), which is a short text with slight information. The data in Indonesian language since Indonesian Twitter users are among the active ones especially during pandemic. This data is not only for validating sentiment and contextual information for stance detection but can also be used for public opinion analysis of vaccination programs that tend to have pros and cons opinion. This dataset adds value aspect-based sentiment information as sub-topics for more accurate sentiment information at the aspect level on tweets, which possibly contains multiple issues discussed. Network features based on interaction relationships were provided for generated user community knowledge. Other researchers may use this data for aspect-based sentiment analysis on tweets to help identify sentiment more accurately, especially on short text.

## 2 Data Description

*There were three data files in our dataset and readme file. All raw data (filtered and unfiltered list of tweets used in this study) are available in the repository Mendeley Data.*

*Data identification number:* 10.17632/7ky2jbjwtn.3

*Direct URL to data*: https://data.mendeley.com/datasets/7ky2jbjwtn/3

  **a.** initial data (Indo_vaccination_raw.csv)

The Initial dataset is a raw data that obtained from the data collection process using Twitter API services. This study collected 2,400,414 Indonesian COVID-19 vaccine-related tweets as the initial dataset (*Indo_vaccination_raw.csv*) during the first ten months of the COVID-19 vaccination program in Indonesia, from January to October 2021. This raw data contains unfiltered list of tweets used in this study.

  **b.** cleaned data (Indo_vaccination_cleaned.csv)

Meanwhile, the cleaned dataset was obtained from the data cleaning and preprocessing. Then the raw dataset was cleaned to remove the irrelevant data, including spam, non-Bahasa (Indonesian language), and non-target related (COVID-19 vaccine) tweets. The duplicated tweets were categorized as spam and removed. Moreover, the data was filtered from a tweet by the government account to represent public opinion without any specific purpose. Finally, 248,604 tweets (representing 10% of the initial dataset) posted by 140,761 unique users on Twitter were cleaned as a raw dataset (*Indo_vaccination_cleaned.csv*).

  **c.** labeled data (*Indo_vaccination_labeled.csv*) dataset

From the cleaned dataset, we conducted data sampling for generated labeled dataset as ground truth, which will be called labeled data. We provided data labeled (*Indo_vaccination_labeled.csv*), collected from the raw dataset, and annotated each tweet with two labeling tasks: stance and aspect-based sentiment. Three independent analysts annotated the sample data with a majority voting strategy for the final label. The labeled data comprised 9,030 tweets posted from 7,313 unique users. The labeled dataset contains six columns (id, user_id, community, aspect_category, aspect_sentiment, stance). All the columns, except community, aspect_category, aspect_sentiment, and stance, are collected using Twitter API services. Meanwhile, the initial and cleaned dataset only contains two columns (id and user_id). Table 1 shows the definition of each column of labeled data.

**Table 1:** Columns of the labeled datasets in the Mendeley database (*Indo_vaccination_labeled.csv*).

Column	Description
*id*	A unique identification number is generated for each Tweet Example: 1376746138108264451
*user_id*	A unique identification number is generated for each Twitter user Example: 508622060
*community*	The community that is user-related (interaction networks) Example: 1
*aspect_category*	Classification of aspect category discussed on Tweet Example: *Implementation*
*aspect_sentiment*	Classification of sentiment that users expressed Example: Negative
*stance*	Classification of the stance that users expressed Example: Against

Due to Twitter's content redistribution policies [1], we only shared Tweet IDs, user IDs, the user community, and the annotation label. Moreover, it also gives users further freedom to use the data. Therefore, researchers should collect the tweets using Twitter API to provide the information needed. Using Louvain modularity, we also provided user community knowledge (column community) extracted from user interaction networks [2]. The interaction network was formed based on mentions and retweet relationships to represent user behavior and agreement. This network can provide contextual information for stance detection tasks [3]. We extracted the data from 1,645 communities, with 88% being communities with less than three members (1,434 communities). The larger communities can number 281 users. Section 2.3 discusses a detailed explanation of user community detection.

The labeled dataset annotations classify three predefined labels of stance [4], including *favor* containing 3,753 tweets, *neutral* with 3,299 tweets, and *against* with 1,978 tweets. Moreover, each tweet is annotated into seven predetermined categories of aspects concerning the target that represent challenges and issues of the COVID-19 vaccination program [5], including *services, implementation, apps, costs, participants, vaccine products*, and *general*. Each tweet can have a multiple-aspect category, with each aspect category having two possible sentiment values: positive and negative. Section 2 explains the details of data labeling, and Table 2 displays the distribution of each class label column. Each aspect category will have two possible sentiment values: positive and negative. Aspect *general* is the most label in our labeled dataset.

**Table 2:** Distribution of each class on data labeled (*Indo_vaccination_labeled.csv*)*.*

Aspect Category	Favor		Against		Neutral		
	Positive	Negative	Positive	Negative	Positive	Negative	Total
*Implementation*	579	276	36	387	437	386	2,101
Services	156	84	2	44	65	53	404
*Costs*	129	36	14	70	62	30	341
*Participants*	350	83	31	178	291	136	1,069
*Apps*	181	88	29	179	183	154	814
*Vaccine products*	622	78	61	328	254	188	1,531
*General*	1,009	278	259	370	825	327	3,068
Total	3,026	923	432	1,556	2,117	1,274	9,328

**Fig. 1:**
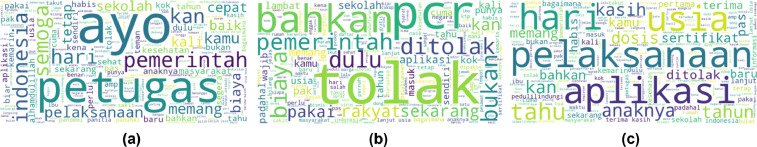
Visualization of the most frequently used keywords in each stance category: (a) in favor, (b) against, (c). neutral.

To provide more detailed information on our data, we visualized each stance category using word clouds in Fig. 1. We obtained these word clouds from the cleaned text after data preprocessing, including normalization, lemmatization, and stopword removal. A detailed explanation of data cleaning and preprocessing is discussed in Section 2. Fig. 1 shows that each category of stance category label has a different characteristic based on word frequency. For example, the high occurrence of the word *ayo* (come on) in the *favor* class indicates an invitation to vaccinate, representing support for the COVID-19 vaccination program. Meanwhile, the *against* class contains several words that express opposition to the COVID-19 vaccination program, including the words *tolak* (reject) and *ditolak* (rejected). Moreover, polymerase chain reaction (PCR) was the phrase with the highest frequency because of the refusal to do the PCR test even though vaccinated. On the other hand, the *neutral* class tends to contain words that represent the category aspect, including *pelaksanaan* (*implementation*), *aplikasi* (application), and *usia* (age). It indicates that the *neutral* class tends not to support or oppose the topic but to ask about an aspect or issue. This is supported by the high number of question marks used in the *neutral* class tweets. Conversely, Table 2 shows that stance and sentiment have orthogonal relationships because negative opinion is not always against the target and vice versa.

**Fig. 2:**
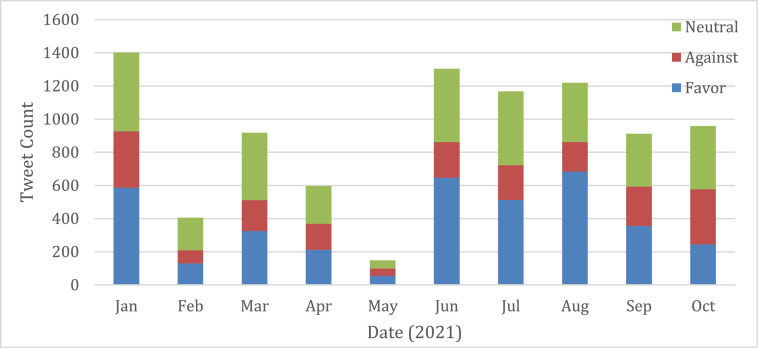
The tweet distribution of our labeled dataset from January to October 2021.

Fig. 2 visualizes the monthly counts of tweets on our labeled dataset. Fig. 2 shows fluctuations, especially between January and June, because several events attract public attention, such as mandatory vaccines for certain administrative requirements in Indonesia. Moreover, the *favor* class dominates every month compared to other classes. We believe this dataset is valuable for many research purposes, especially public opinion analysis on Twitter, including stance detection, aspect-based sentiment analysis, and social network analysis. On the other hand, there is skepticism regarding vaccines in several social groups [6], which makes it interesting to use as a case study, especially in Indonesia, which has ethnic and religious diversity [7]. Therefore, a public opinion analysis on COVID-19 vaccination is needed as public health surveillance to prevent rejection and increase public acceptance of the COVID-19 vaccine.

## 3 Experimental Design, Materials and Methods

### 3.1 Data Collection and Preprocessing

The data were collected using Twitter API^1^ on Python with specific keywords: *vaksin* (vaccines) and *vaksinasi* (vaccination). The tweets used are Indonesian tweets posted between January and October 2021, Indonesia's first period of COVID-19 vaccination. We collected a maximum of 250,000 sample tweets each month to overcome the limitations of Twitter API services. This process collects the text of a tweet and other related information, including Twitter metrics and user metadata. There were 2,400,414 Indonesian COVID-19 vaccine-related tweets from 576,488 Twitter users (*Indo_vaccination_raw.csv*) as the initial dataset. The study selected Indonesian tweets based on the attribute language “in” on Twitter API services. However, we found a lot of irrelevant data, including non-Bahasa (Indonesian language), non-COVID-19 vaccine-related, and spam tweets, so we conducted data preprocessing and cleaning to remove the irrelevant data for the cleaned dataset.

The first step in data preprocessing was case folding to map the text to lowercase format. The irrelevant Twitter attributes were removed, including user mentions, hashtags, numbers, symbols, and emojis. The text of tweets generally contains slang words. Therefore, text normalization was carried out based on the Indonesian slang corpus^2^. Further data preprocessing includes lemmatization using *NLP-ID*^3^ and removing the stopwords using *Sastrawi*^4^. After the data preprocessing was complete, data cleaning was carried out, thus removing the irrelevant data (such as non-Bahasa, non-COVID-19 vaccine-related, and spam tweets). We found that in our initial dataset, there are many Malaysian tweets, even though it has been selected based on the Twitter attribute. Therefore, we filtered non-Bahasa (Malaysian tweets) and non-COVID-19 vaccine-related tweets based on the collected keywords.

**Fig. 3:**
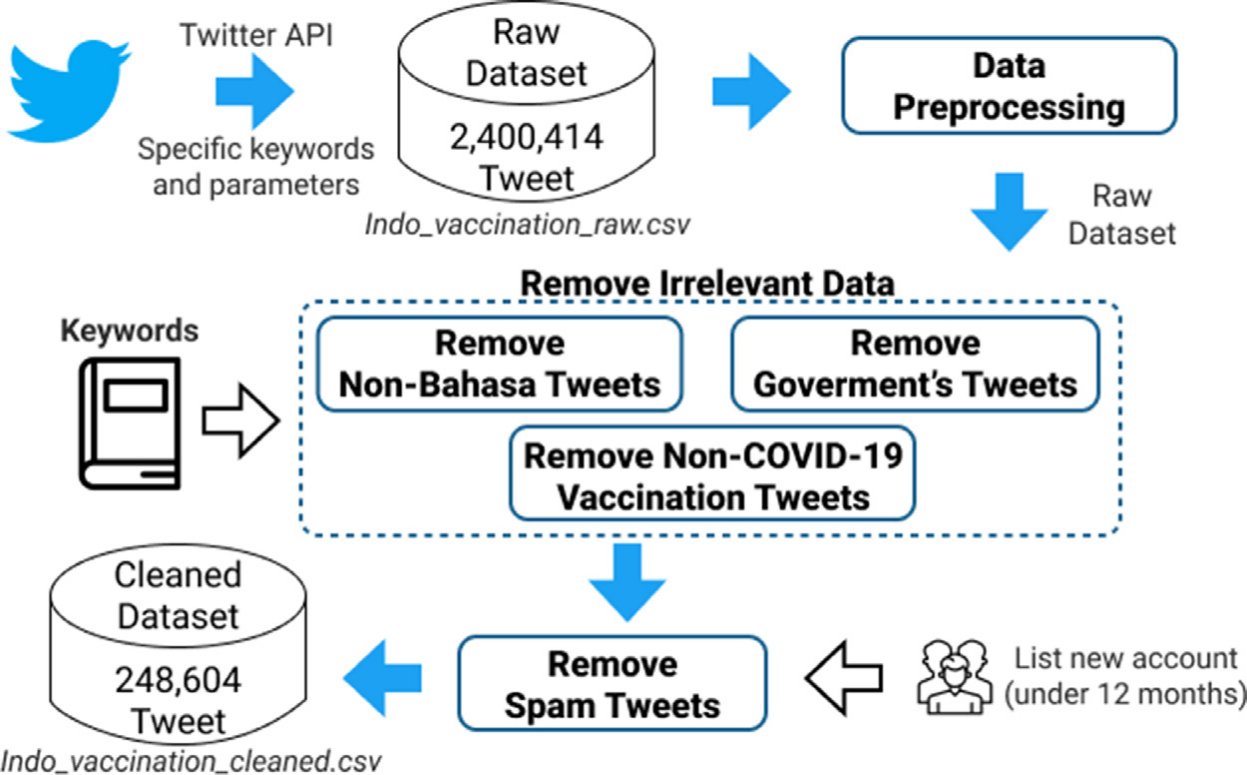
Methodology of our data collection and preprocessing.

Moreover, we removed a tweet posted by the government to prevent bias in data; the government tends to support the COVID-19 vaccination policy and continuously posted duplicate tweets to exaggerate information (spam tweets). We also only used accounts over 12 months since November 2021 because there is a possibility that a new Twitter account is a spammer. Finally, we obtained the cleaned dataset of 248,604 posts from 140,761 users (*Indo_vaccination_cleaned.csv*), representing 10% of the initial dataset (*Indo_vaccination_raw.csv*). Fig. 3 describes our methodology for data collection and preprocessing.

### 3.2 Data Labeling

After obtaining the cleaned dataset, we conducted data sampling for labeling as ground truth. Data sampling was conducted based on keywords collected manually, representing each stance class. The sample data includes 9,030 manually selected tweets from the cleaned dataset (*Indo_vaccination_labeled.csv*), which were identified by two labeling tasks: stance and aspect-based sentiment labels. Three independent analysts—two researchers in natural language processing (one MSc-level and one BSc-level) and one communication science expert (BS*c*-level)—labeled the data manually and used the majority voting strategy for the final class label. We only used unique tweets to provide reliable labeled data and did not consider the retweets. This study applied data labeling using LabelStudio^5^*.* For stance labeling, each tweet was annotated into three classes: *favor, against,* and *neutra*l. The tweet was given an *favo*r or *against* label if the opinion supported or opposed the target. The tweet was labeled *neutral* if the opinion was neither of these two cases or the statement about the target was inconclusive.

The study annotated each tweet into six predetermined aspects for the aspect-based sentiment labeling, including *services, implementation, apps, costs, participants*, and *vaccine products*. These predetermined aspects represent the challenges and issues of the COVID-19 vaccination program on Twitter [5,6], and particularly in Indonesia [8]. In addition, the vaccine product influences the acceptance of the COVID-19 vaccine in Indonesia [9]. However, there is a possibility that the user expresses an opinion without stating any of the predetermined aspects [10]. Therefore, this opinion was categorized as a *general* aspect. In total, we used seven aspects. Each aspect has two possible sentiment values, positive or negative, for simple binary polarities. There is a possibility that COVID-19 vaccine-related tweets may contain more than one aspect or subtopic, discussed with different sentiments. If a tweet had multi-aspects, the sentiments of the aspects were categorized into one-sentiment information. Therefore, if a tweet has different sentiments between aspects, its sentiment label is conflict. Table 3 illustrates the mechanism to determine the sentiment label of tweets. In Ex. (1) in Table 3, the analyst labeled a tweet with three aspects (multi-aspect) with positive sentiments that dominate among aspects. Therefore, the sentiment is positive by voting the sentiment label on the tweet. Unlike Ex. (2) in Table 3, aspects' sentiments differ. Therefore, the sentiment is categorized as conflict.

**Table 3:** The mechanism to determine the final sentiment label by annotator. A case study on aspect-based sentiment labeling.

Ex.	Tweet (in Bahasa)		Label by Annotator		
		English Translation	Aspect-based Sentiment	Aspect Category	Sentiment
(1)	*Vaksinasi gratis tersebut dilaksanakan dengan tujuan untuk mempercepat program vaksinasi Covid-19 pada masyarakat.*	The free vaccination was conducted to accelerate the Covid-19 vaccination program in society	Implementation – Positive Cost - Positive	Implementation, Cost	Positive
(2)	*@__Sridiana_3va Vaksin kan katanya gratis, dan yang belum saya fahami kenapa pelaksanaan vaksin itu bisa diadakan oleh partai atau lembaga? Mereka hanya ketempatan lokasi atau bagaimana? Kenapa harus disana?*	@__Sridiana_3va Vaccines are said to be free, and I do not understand why parties or institutions can implement the vaccine? Are they just local locations or what? Why should it be there? □	Implementation – Negative Cost - Positive	Implementation, Cost	Conflict

We applied the majority voting strategy to the final label of the tweet. Thus, if each analyst's notation differs, the tweet is marked as class *invalid*. Table 4 shows an example of our majority voting strategy. In Ex. (1) in Table 4, the final label voted is *cost* because two analysts labeled it as c*ost*. Unlike Ex. (2) in Table 4, the analysts' labels differed. Therefore, they categorized the final label as *invalid*. This approach also applied to the vote of the stance category label. We calculated Cohen's kappa coefficient for stance and aspect-based sentiment labels to evaluate the agreement between analysts. We obtained a moderate consensus among the analysts, with *Cohen's kappa* coefficients of 0.6517 for the stance and *Krippendorff's Alpha* of 0.5187 for the aspect-based sentiment label. This was necessary because the analysts have different points of view, especially regarding the *positive* and *neutral* classes, which tend to be challenging to identify. In aspect-based sentiment labeling, the analysts categorized the aspects discussed differently due to the lack of context in the text of the tweet.

**Table 4:** The mechanism of our majority voting strategy. A case study on aspect category label.

Ex.	Tweet (in Bahasa)	English Translation	Annotator			Final Label
			A	B	C	
(1)	*Mereka memaki orangnya, tapi uang BLT dimakan juga. Bilang bansos dimaling, dia ngaku rakyat miskin yg berhak bansos. Ngaku orang kaya, minta vaksin gratis. Tolak vaksin cina, ngarep yg jerman (kapir kapir juga kan?)**ya itulah kadrun*	They cursed the other person, but the BLT aid was obtained too. He said that social assistance was stolen but admitted that the poor were entitled to social service. Claiming to be rich people asking for free vaccines. Reject the Chinese vaccine, hoping for the German one (also pagans, right?) yeah, that's Kadrun	General	Participants	Vaccine products	Invalid
(2)	*@collegemenfess Vaksin itu program pemerintah yang gratis nder jadi ayo vaksin*	@collegemenfess Vaccination is a free government program, so let's get vaccinated	Cost	Cost	General	Cost

### 3.3 Community Detection

Since network features can be contextual information for detecting peoples' stances [3], we also provided user community knowledge. Twitter network features can be divided into three categories based on how to formulate, including preference, interaction, and connection networks [4]. This study used an interaction network based on mention and retweet relationships. This process is performed on the cleaned data to represent the user network better. First, we extracted the mentioned relationship based on the prefix "@." At the same time, the term "RT" at the beginning of a tweet extracted the retweet relationship. The combined labeled data contains 4,755 retweets and 9,247 mentions of relationships. Then the combined relations implemented community detection using the Louvain modularity algorithm.

**Fig. 4:**
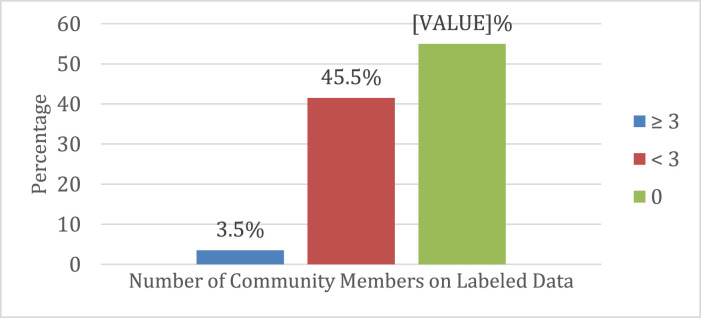
Comparisons of community members on labeled data.

**Fig. 5:**
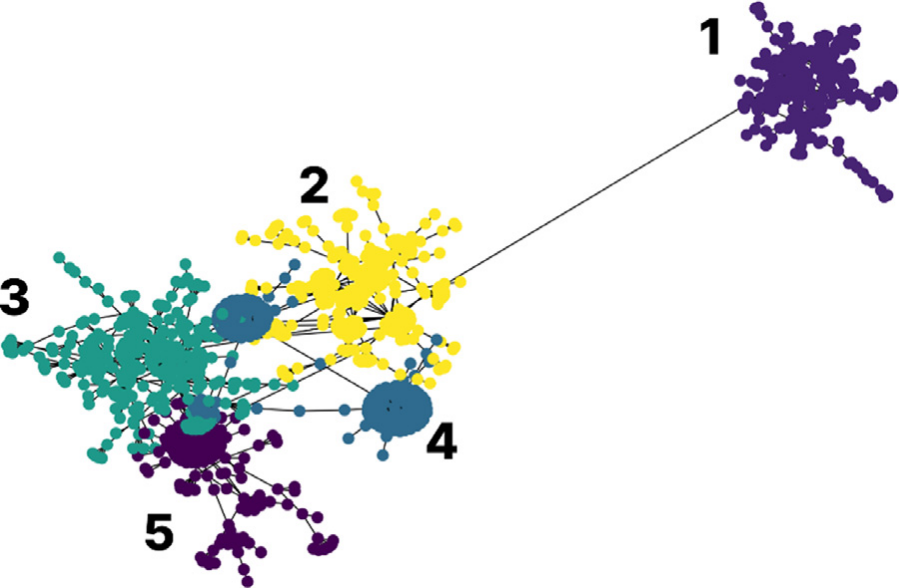
Graph of the five largest communities with the most members.

We implemented Louvain modularity using networkX^6^ with default parameters. There were communities of 3,648 that contained edges of 9,840 (representing relationships) and nodes of 10,299 (representing Twitter users). We only used users that posted in our labeled data. However, some communities have a small number of members. Therefore, we based our selection on the minimum number of community members threshold. In this study, we used three users as the threshold of community members because 96.5% of the community has less than three members. There are 130 (representing 3.5%) of the communities that are valid (with at least three members) in the labeled data. Fig. 4 shows the comparisons of communities detected on labeled data. To demonstrate the community extracted, we visualize the graphs of the five largest communities by the number of members in Fig. 5. There are 55% communities do not have member on our labeled data. Meanwhile, 45.5% communities only have one or two members on a community.

Based on Fig. 5, there are five communities with a large number of members. Based on the class label, communities two and five classify *favor* and *neutral*, while community four classify *favor* and *agains*t COVID-19 vaccination. Meanwhile, community one tends to be *favor,* and community three tends to be *against* COVID-19 vaccination. No particular aspects were discussed in the community, except for community one regarding vaccines and community five regarding application aspects. It indicates that community information is not always related to the same issues or discussed aspects. Community ‘B’ and ‘E’ tend to be class Favor and Neutral, while community ‘D’ tends to be class Favor and Against COVID-19 Vaccination. Meanwhile, community ‘A’ tends to be class Favor and community ‘C’ tends to be against COVID-19 vaccination.

### 3.4 Preliminary Experiment

We also evaluated our data using classification models to demonstrate the potential and quality of our data set for the stance detection task. We applied five machine learning and four sequential-based deep learning models. Five machine-learning models, including Naïve Bayes (NB), K-Nearest Neighbor (KNN), Decision Tree (DT), Support Vector Machine (SVM), and Random Forest (RF), were implemented using sklearn^7^ with default parameters. Meanwhile, four deep learning models, including Gated Recurrent Unit (GRU), Bidirectional GRU (BiGRU), Long Short-Term Memory (LSTM), and Bidirectional LSTM (BiLSTM), were applied using Keras^8^ and adopted modest neural network architecture [11]. In our experiment, we used tweets' text opinions, aspect-based sentiment information, and user community knowledge as features for stance detection. We used cleaned text, the result from the previous section (Section 2.1), then used TF-IDF for machine learning and a word-embedding model for deep learning as vector representation. Aspect-based sentiment information, including aspect category and aspect sentiment, was labeled (Section 2.2) and decomposed using a binary relevance approach.

The study applied Word2Vec as a word-embedding model as text representations for machine learning and deep learning models. We used pre-trained word embeddings that trained on 467,000 documents of Indonesian Wikipedia for 300-dimensional word representation. Each model was evaluated using 5-fold cross-validation to use 80% for learning and 20% of labeled data for testing the model. The result shows that RF with TF-IDF as a text representation outperformed other classifiers with an average accuracy of 58.1% and a Macro F1 score of 57.1%, as shown in Table 5. Meanwhile, BiLSTM obtained the best performance of deep learning models with an average accuracy of 57.3% and a Macro F1 score of 55.9%. However, the result shows that deep learning models consistently perform well.

**Table 5:** Classification performance for nine classifiers using 5-fold cross-validation.

Classifier	Text Representation	Accuracy	Macro Precision	Macro Recall	Macro F1-Score
NB	TF-IDF	52.9	59.5	46.2	44.8
KNN		53.9	53.4	52.4	52.1
DT		51.2	49.8	49.9	49.8
RF		58.1	58.5	56.9	57.1
SVM		56.5	55.9	55.2	55.5
GRU	Word2Vec	56.5	57.2	53.3	54.3
LSTM		56.3	55.9	54.0	54.1
BiLSTM		57.3	56.5	56.4	55.9
BiGRU		57.0	56.4	55.9	55.4

### 3.5 Discussion

There are nine classifiers implemented as the preliminary experiment of our labeled dataset. The result is provided in Table 5 and shows that our dataset is adequate for stance detection modeling, especially in a low-resource language like Indonesian. Surprisingly, Random Forest with TF-IDF as text representation based on term frequency obtained a better result on all evaluation metrics than deep learning models. The BiLSTM achieved the next best performance with Word2Vec as text representation with an accuracy of 57.3%. Moreover, deep learning tends to be more consistent than machine learning, in which only two models obtain more than 55% accuracy. However, the features used are still modest and do not adequately represent the stance. Stance detection performs well if it uses several features, such as linguistics, social interaction, and user identity [3,4].

On the other hand, a tweet is a short text and lacks contextual information. Therefore, features representing contextual information are necessary to improve the stance detection performance. Despite using *general*-purpose classification methods rather than state-of-the-art stance detection models, all models could classify the tweets with acceptable performance. For further study, researchers can use our dataset for public opinion analysis to understand the public stance and sentiment during the COVID-19 pandemic, especially in Indonesia.

## Ethics Statements

Our data were collected, scraped, and distributed under the Twitter developer policy 2022 and followed its regulations [1]. Data from Twitter could be used with care for the Privacy and Control of Twitter users. In the discussion, we processed the data and did not mention the privacy information to protect Twitter users and ensure anonymity.

## CRediT authorship contribution statement

**Diana Purwitasari:** Conceptualization, Methodology, Supervision, Funding acquisition, Writing – review & editing, Project administration. **Cornelius Bagus Purnama Putra:** Conceptualization, Methodology, Software, Writing – original draft, Data curation, Investigation. **Agus Budi Raharjo:** Writing – review & editing.

## Declaration of Competing Interest

The authors declare that they have no known competing financial interests or personal relationships that could have appeared to influence the work reported in this paper.

## Data Availability

Indonesian COVID-19 Vaccination-related Tweets for Stance Detection and Aspect-based Sentiment Analysis (Original data) (Mendeley Data)

## Footnotes

1 https://developer.twitter.com/en/docs/twitter-api/tweets/search/api-reference/get-tweets-search-all.

2 https://github.com/louisowen6/NLP_bahasa_resources/blob/master/combined_slang_words.txt.

3 https://github.com/kumparan/nlp-id.

4 https://github.com/har07/PySastrawi.

5 https://labelstud.io/.

6 *https://networkx.org/*.

7 *https://scikit-learn.org/*.

8 *https://keras.io/*.
